# Ge/Si(001) heterostructures with dense arrays of Ge quantum dots: morphology, defects, photo-emf spectra and terahertz conductivity

**DOI:** 10.1186/1556-276X-7-414

**Published:** 2012-07-23

**Authors:** Vladimir A Yuryev, Larisa V Arapkina, Mikhail S Storozhevykh, Valery A Chapnin, Kirill V Chizh, Oleg V Uvarov, Victor P Kalinushkin, Elena S Zhukova, Anatoly S Prokhorov, Igor E Spektor, Boris P Gorshunov

**Affiliations:** 1 A M Prokhorov General Physics Institute of RAS, 38 Vavilov Street, Moscow, 119991, Russia; 2 Technopark of GPI RAS, 38 Vavilov Street, Moscow, 119991, Russia; 3 Moscow Institute of Physics and Technology, Institutsky Per. 9, Moscow Region, 141700, Dolgoprudny, Russia

## Abstract

Issues of Ge hut cluster array formation and growth at low temperatures on the Ge/Si(001) wetting layer are discussed on the basis of explorations performed by high resolution STM and *in-situ* RHEED. Dynamics of the RHEED patterns in the process of Ge hut array formation is investigated at low and high temperatures of Ge deposition. Different dynamics of RHEED patterns during the deposition of Ge atoms in different growth modes is observed, which reflects the difference in adatom mobility and their ‘condensation’ fluxes from Ge 2D gas on the surface for different modes, which in turn control the nucleation rates and densities of Ge clusters. Data of HRTEM studies of multilayer Ge/Si heterostructures are presented with the focus on low-temperature formation of perfect films.

Heteroepitaxial Si *p–i–n*-diodes with multilayer stacks of Ge/Si(001) quantum dot dense arrays built in intrinsic domains have been investigated and found to exhibit the photo-emf in a wide spectral range from 0.8 to 5 *μ*m. An effect of wide-band irradiation by infrared light on the photo-emf spectra has been observed. Photo-emf in different spectral ranges has been found to be differently affected by the wide-band irradiation. A significant increase in photo-emf is observed in the fundamental absorption range under the wide-band irradiation. The observed phenomena are explained in terms of positive and neutral charge states of the quantum dot layers and the Coulomb potential of the quantum dot ensemble. A new design of quantum dot infrared photodetectors is proposed.

By using a coherent source spectrometer, first measurements of terahertz dynamical conductivity (absorptivity) spectra of Ge/Si(001) heterostructures were performed at frequencies ranged from 0.3 to 1.2 THz in the temperature interval from 300 to 5 K. The effective dynamical conductivity of the heterostructures with Ge quantum dots has been discovered to be significantly higher than that of the structure with the same amount of bulk germanium (not organized in an array of quantum dots). The excess conductivity is not observed in the structures with the Ge coverage less than 8 Å. When a Ge/Si(001) sample is cooled down the conductivity of the heterostructure decreases.

## Introduction

Artificial low-dimensional nano-sized objects, like quantum dots, quantum wires and quantum wells, as well as structures based on them, are promising systems for improvement of existing devices and for development of principally new devices for opto-, micro- and nano-electronics. Besides, the investigation of physical properties of such structures is also of fundamental importance. In both regards, amazing perspectives are provided when playing around with quantum dots that can be considered as artificial atoms with a controlled number of charge carriers that have a discrete energy spectrum [1,2]. Arrays of a *large* number of quantum dots including multilayer heterostructures make it possible to create artificial “solids” whose properties can be controllably changed by varying the characteristics of constituent elements (“atoms”) and/or the environment (semiconductor matrix). The rich set of exciting physical properties in this kind of systems originates from single-particle and collective interactions that depend on the number and mobility of carriers in quantum dots, Coulomb interaction between the carriers inside a quantum dot and in neighbouring quantum dots, charge coupling between neighbouring quantum dots, polaron and exciton effects, etc. Since characteristic energy scales of these interactions (distance between energy levels, Coulomb interaction between charges in quantum dots, one- and multiparticle exciton and polaron effects, plasmon excitations, etc.) are of order of several meV [3-5], an appropriate experimental tool for their study is provided by optical spectroscopy in the far-infrared and terahertz bands.

To get access to the effects, one has to extend the operation range of the spectrometers to the corresponding frequency domain that is to the terahertz frequency band. Because of inaccessibility of this band, and especially of its lowest frequency part, below 1 THz (that is ≲33 cm^−1^), for standard infrared Fourier-transform spectrometers, correspondent data is presently missing in the literature. In this paper, we present the results of the first detailed measurements of the absolute dynamical (AC) conductivity of multilayer Ge/Si heterostructures with Ge quantum dots, at terahertz and sub-terahertz frequencies and in the temperature range from 5 to 300 K.

In addition, for at least two tens of years, multilayer Ge/Si heterostructures with quantum dots have been candidates to the role of photosensitive elements of monolithic IR arrays promising to replace and excel platinum silicide in this important brunch of the sensor technology [6-8]. Unfortunately, to date achievements in this field have been less than modest.

We believe that this state of affairs may be improved by rigorous investigation of formation, defects and other aspects of materials science of such structures, especially those which may affect device performance and reliability, focusing on identification of reasons of low quantum efficiency and detectivity, high dark current and tend to degrade with time as well as on search of ways to overcome these deficiences. New approaches to device architecture and design as well as to principles of functioning are also desirable.

This article reports our latest data on morphology and defects of Ge/Si heterostructures. On the basis of our recent results on the photo-emf in the Si *p–i–n*-structures with Ge quantum dots, which are also reported in this article, we propose a new design of photovoltaic quantum dot infrared photodetectors.

## Methods

### Equipment and techniques

The Ge/Si samples were grown and characterized using an integrated ultrahigh vacuum instrument [9-12] built on the basis of the Riber SSC 2 surface science center with the EVA 32 molecular-beam epitaxy (MBE) chamber equipped with the RH20 reflection high-energy electron diffraction (RHEED) tool (Staib Instruments) and connected through a transfer line to the GPI-300 ultrahigh vacuum scanning tunnelling microscope (STM) [13-15]. Sources with the electron beam evaporation were used for Ge or Si deposition. A Knudsen effusion cells was utilized if boron doping was applied for QDIP *p–i–n*-structure formation. The pressure of about 5×10^−9^ Torr was kept in the preliminary sample cleaning (annealing) chamber. The MBE chamber was evacuated down to about 10^−11^ Torr before processes; the pressure increased to nearly 2×10^−9^ Torr at most during the Si substrate cleaning and 10^−9^ Torr during Ge or Si deposition. The residual gas pressure did not exceed 10^−10^ Torr in the STM chamber. Additional details of the experimental instruments and process control can be found in Ref. [12].

RHEED measurements were carried out *in situ*, i.e., directly in the MBE chamber during a process [10]. STM images were obtained in the constant tunnelling current mode at the room temperature. The STM tip was zero-biased while the sample was positively or negatively biased when scanned in empty- or filled-states imaging mode. Structural properties of the Ge/Si films were explored by using the Carl Zeiss Libra-200 FE HR HRTEM.

The images were processed using the WSxM software [16].

For obtaining spectra of photo-electromotive force (photo-emf) a setup enabling sample illumination by two independent beams was used; one of the beams was a wide-band infrared (IR) radiation, generated by a tungsten bulb, passed through a filter of Si or Ge (bias lighting) and the other was a beam-chopper modulated narrow-band radiation cut from globar emission by an IR monochromator tunable in the range from 0.8 to 20 *μ*m. The spectra were taken at the chopping frequency of 12.5 Hz at temperatures ranged from 300 to 70 K and a widely varied power of the bias lighting.

The measurements of the terahertz dynamic conductivity and absorptivity of Ge/Si heterostructures at room and cryogenic temperatures (down to 5 K) have been performed using the spectrometer based on backward-wave oscillators (BWO) as radiation sources. This advanced experimental technique will be described in detail below in a separate section.

### Sample preparation procedures

#### Preparation of samples for STM and RHEED

Initial samples for STM and RHEED studies were 8×8 mm^2^ squares cut from the specially treated commercial boron-doped Czochralski-grown (CZ) Si(100) wafers (*p*-type, *ρ*=12 Ω cm). After washing and chemical treatment following the standard procedure described elsewhere [17], which included washing in ethanol, etching in the mixture of HNO_3_ and HF and rinsing in the deionized water [12], the silicon substrates were loaded into the airlock and transferred into the preliminary annealing chamber where they were outgassed at the temperature of around 565℃ for more than 6 h. After that, the substrates were moved for final treatment and Ge deposition into the MBE chamber where they were subjected to two-stages annealing during heating with stoppages at 600℃ for 5 min and at 800℃ for 3 min [9,10]. The final annealing at the temperature greater than 900℃ was carried out for nearly 2.5 min with the maximum temperature of about 925℃ (1.5 min). Then, the temperature was rapidly lowered to about 750℃. The rate of the further cooling was around 0.4℃/s that corresponded to the ‘quenching’ mode applied in [10]. The surfaces of the silicon substrates were completely purified of the oxide film as a result of this treatment [10,18,19].

Ge was deposited directly on the deoxidized Si(001) surface. The deposition rate was varied from about 0.1 to 0.15 Å/s; the effective Ge film thickness (*h*_Ge_) was varied from 3 to 18 Å for different samples. The substrate temperature during Ge deposition (*T*_gr_) was 360℃ for the low-temperature mode and 600 or 650℃ for the high-temperature mode. The rate of the sample cooling down to the room temperature was approximately 0.4℃/s after the deposition.

#### Preparation of multilayer structures

Ge/Si heterostructures with buried Ge layers were grown on CZ *p*-Si(100):B wafers (*ρ* =12 Ω cm) washed and outgassed as described above. Deoxidized Si(001) surfaces were prepared by a process allowed us to obtain clean substrate surfaces (this was verified by STM and RHEED) and perfect epitaxial interfaces with Si buffer layers (verified by HRTEM): the wafers were annealed at 800℃ under Si flux of ≲0.1 Å/s until a total amount of the deposited Si, expressed in the units of the Si film thickness indicated by the film thickness monitor, reached 30 Å; 2-minute stoppages of Si deposition were made first twice after every 5 Å and then twice after every 10 Å.

Afterwards, a ∼ 100 nm thick Si buffer was deposited on the prepared surface at the temperature of ∼ 650℃. Then, a single Ge layer or a multilayer Ge/Si structure was grown. A number of Ge layers in multilayer structures reached 15 but usually was 5; their effective thickness (*h*_Ge_), permanent for each sample, was varied from sample to sample in the range from 4 to 18 Å; the thickness of the Si spacers (*h*_Si_) was ∼ 50 nm. The Ge deposition temperature was ∼ 360℃, Si spacers were grown at ∼ 530℃. A heterostructure formed in such a way was capped by a ∼ 100 nm thick Si layer grown at ∼ 530℃. All layers were undoped.

The samples were quenched after the growth at the rate of ∼ 0.4℃/s.

#### Growth of *p–i–n*-structures

*p–i–n*-structures were grown on commercial phosphorus-doped CZ *n*-Si(100) substrates (*ρ*=0.1 Ω cm). Si surfaces were prepared for structure deposition in the same way as for the growth of multilayer structures. *i*-Si buffer domains of various thicknesses were grown on the clean surfaces at ∼ 650℃. Then, a stacked structure of several periods of quantum dot (QD) dense arrays separated by Si barriers was grown under the same conditions as the multilayer structures; *h*_Si_ was widely varied in different structures reaching 50 nm; *h*_Ge_ always was 10 Å. A sufficiently thick undoped Si layer separated the stacked QD array from the Si:B cap doped during the growth, the both layers were grown at ∼ 530℃.

**Figure 1:**
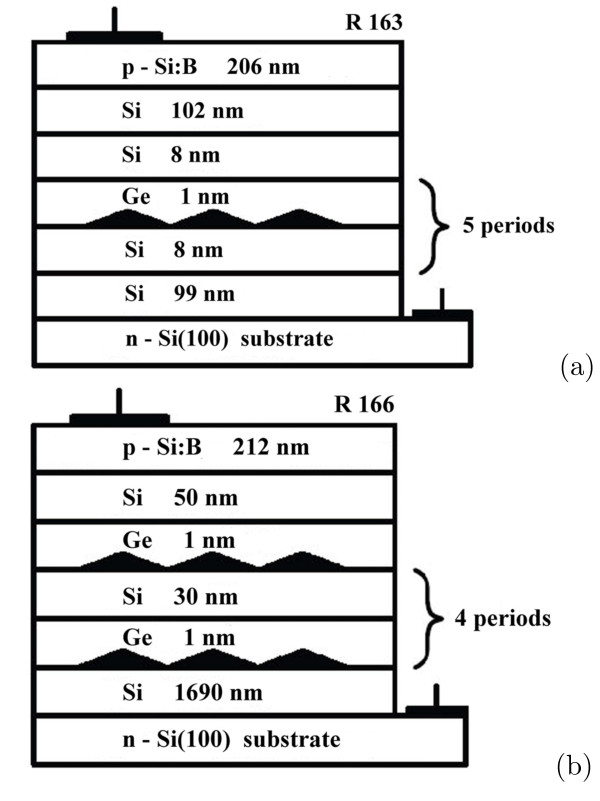
**Schematics of the *p–i–n*-structures.** Stacks of five layers of Ge hut clusters (quantum dots) separated by Si barriers are built in the intrinsic domains of the *p–i–n* diodes; the *p–i–n* mesa structures were formed on CZ n-Si(100) substrates (*ρ*=0.1 Ω cm); composition and thickness of each layer of the mesa structures as well as locations of Al contacts are shown in the scatches; **(a)** in the R 163 structure, the boron concentration in the Si cap layer is ∼5×10^18^ cm^−3^; **(b)** the boron concentration in the Si cap layer of the R 166 structure is ∼10^19^ cm^−3^.

Figure 1 demonstrates two such structures (referred to as R 163 and R 166) which are in the focus of this article. Their caps were doped to 5×10^18^ and 10^19^ cm^−3^ in the R 163 and R 166 samples, respectively. Buffer layer and barrier thicknesses were 99 and 8 nm in the R 163 structure and 1690 and 30 nm in R 166.

Mesas were formed on samples for photoelectric measurements. Ohmic contacts were formed by thermal deposition of aluminum.

### Terahertz BWO-spectroscopy

The BWO-spectrometers provide broad-band operation (frequencies *ν* ranging from 30 GHz to 2 THz), high frequency resolution (*Δν*/*ν*=10^−5^), broad dynamic range (40–50 dB), continuous frequency tuning and, very importantly, the possibility of *direct* determination of spectra of any “optical” parameter, like complex conductivity, complex dielectric permittivity, etc. (‘direct’ means that no Kramers–Kronig analysis—typical for far-infrared Fourier transform spectroscopy—is needed). The principle of operation of BWO-spectrometers is described in details in the literature (see, e.g., [20,21]). It is based on measurement of the complex transmission coefficient $Tr^{*}=\text{Tr}\exp(\mathrm{i\varphi})$ of a plane-parallel sample with subsequent calculation of the spectra of its optical parameters from those of the transmission coefficient amplitude *Tr*(*ν*) and the phase *φ*(*ν*). The corresponding expression can be written as [22,23]

(1) $$Tr^{*}=\text{Tr}\exp(\mathrm{i\varphi})=\frac{T_{12}T_{21}\exp(\mathrm{i\delta})}{1+T_{12}T_{21}\exp(2\mathrm{i\delta})}.$$

Here

(2) $$\begin{array}{cc} T_{\text{pq}} & =t_{\text{pq}}\exp(i\varphi_{\text{pq}}),t_{\text{pq}}^{2}=\frac{4\left(n_{p}^{2}+k_{p}^{2}\right)}{{\left(k_{p}+k_{q}\right)}^{2}+{\left(n_{p}+n_{q}\right)}^{2}},\\ \varphi_{\text{pq}} & =\arctan\left\{\frac{k_{p}n_{p}-k_{q}n_{q}}{\overset{2}{\underset{p}{n}}+k_{p}^{2}+n_{p}n_{q}+k_{p}k_{q}}\right\} \end{array}$$

are Fresnel coefficients for the interfaces ‘air–sample’, indices *p*, *q*=1, 2 correspond: ‘1’ to air (refractive index *n*_1_=1, extinction coefficient *k*_1_=0) and ‘2’ to the material of the sample (*n*_2_, *k*_2_), $\delta =\frac{2\mathrm{\pi d}}{\lambda }(n_{2}+ik_{2})$*d* is the sample thickness, *λ* is the radiation wavelength. The sample parameters (for instance, *n*_2_ and *k*_2_ ) are found for each fixed frequency by solving two coupled equations for the two unknowns, *Tr*(*n*_2_, * k*_2_,* ν*)=*Tr*_exp_(*ν*) and *φ*(*n*_2_,* k*_2_,* ν*)=*φ*_exp_(*ν*) [here *Tr*_exp_(*ν*) and *φ*_exp_(*ν*) are the measured quantities]. The so-found values of *n*_2_(*ν*) and *k*_2_(*ν*) can then be used to derive the spectra of the complex permittivity $\varepsilon^{*}(\nu )=\varepsilon^{\prime }(\nu )+i\varepsilon^{\prime \prime }(\nu )=n_{2}^{2}-k_{2}^{2}+2in_{2}k_{2}$, complex conductivity $\sigma^{*}(\nu )=\sigma_{1}(\nu )+i\sigma_{2}(\nu )=\nu n_{2}k_{2}+\mathrm{i\nu}(\varepsilon_{\infty }-\varepsilon^{\prime })/2$, etc. ( $\varepsilon_{\infty }$ is the high-frequency contribution to the permittivity).

If the sample is characterized by low enough absorption coefficient, Fabry–Perot-like interference of the radiation within the plane-parallel layer leads to an interference maxima and minima in the transmission coefficient spectra. In this case there is no need to measure the phase shift spectra since the pairs of optical quantities of the sample can be calculated from the transmission coefficient spectrum alone: the absorptive part (like *ε*^′^ or *σ*_1_) is determined from the interferometric maxima amplitudes and the refractive part (like *ε*^*′*^ or *n*) is calculated from their positions [20,21].

When measuring the dielectric response of the films (like heterostructures in the present case) on dielectric substrates, first the dielectric properties of the substrate material are determined by standard techniques just described. Next, one measures the spectra of the transmission coefficient and of the phase shift of the film-substrate system, and it is these spectra that are used to derive the dielectric response of the film by solving two coupled equations for two unknowns—“optical” parameters of the film. The corresponding expression for the complex transmission coefficient of a two-layer system can be written as [22,23]:

(3) $$\begin{array}{cc} \;Tr_{1234}^{*} & =\text{Tr}\exp(\mathrm{i\varphi})\\ =\frac{T_{12}T_{23}T_{34}\exp\{i(\delta_{2}+\delta_{3})\}}{1\;+\;T_{23}T_{34}\exp(2i\delta_{3})\;+\;T_{12}T_{23}\exp(2i\delta_{2})\;+\;T_{12}T_{34}\exp\{2i(\delta_{2}\;+\;\delta_{3})\}}, \end{array}$$

where indices 1 and 4 refer to the media on the two sides of the sample, i.e., of the film on substrate, *δ*_*p*_=(*n*_*p*_ + *ik*_*p*_), with *d*_*p*_ being the film and substrate thicknesses (*p*=2, 3). The other notations are the same as in Eq. (1). The measurements are performed in a quasioptical configuration, no waveguides are used [20,21] and this makes measurement schemes extremely flexible. All measurement and analysis procedures are PC-controlled. Most important parameters of the BWO-spectrometer are summarized in Table 1.

**Table 1:** Main parameters of the terahertz BWO-spectrometer

Frequency range , THz	0.03 to 2
Probing radiation power, mW	1 to 100
Frequency resolution:	
relative, *Δν*/*ν*	10^−4^–10^−5^
absolute, cm^−1^	0.001
Dynamical range, dB	40 to 60
Signal to noise ratio	10^4^ to 10^6^
Probing radiation polarization degree, %	99.99
Time to record a spectrum of 100 points, s:	
amplitude *Tr*(*ν*)	10 to 20
phase *φ*(*ν*)	20 to 40
Temperature interval, K	2 to 300
Magnetic fields, T	up to 7

## Results and discussion

### Morphology and defects

#### STM and RHEED study of Ge/Si(001) QD arrays: morphology and formation

**Figure 2:**
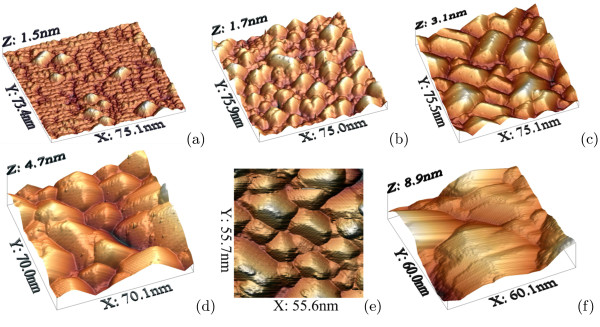
**STM images of Ge/Si(001) quantum dot arrays grown at 360℃. ***h*_Ge_(Å) is (**a**) 6, (**b**) 8, (**c**) 10, (**d**) 14, (**e**) 15, (**f**) 18.

Previously, we have shown in a number of *STM studies*[9,11,12,24-26] that the process of the hut array nucleation and growth at low temperatures starts from occurrence of two types of 16-dimer nuclei [25] on wetting layer (WL) patches of 4-ML height [26] giving rise to two known species of {105}-faceted clusters—pyramids and wedges [9]—which then, growing in height (both types) and in length (wedges), gradually occupy the whole wetting layer, coalless and start to form a nanocrystalline Ge film (Figure 2) [11,12]. This is a life cycle of hut arrays at the temperatures < 600℃. We refer to cluster growth at these temperatures as the low-temperature mode.

At high temperatures (> 600℃), only pyramids represent a family of huts: they were found to nucleate on the WL patches in the same process of 16-dimer structure occurrence as at low temperatures [24]. We failed to find wedges or their nuclei if Ge was deposited at these temperatures and this fact waits for a theoretical explanation.

In addition to pyramids, shapeless Ge heaps faceting during annealing have been observed on WL in the vicinity of pits and interpreted as possible precursors of large faceted clusters [12,24]. Note that a mechanism of Ge hut formation via faceting of some shapeless structures appearing near WL irregularities, which resembles the process described in the current article, was previously considered as the only way of Ge cluster nucleation on Si(001) [27,28]. Now we realize that huts nucleate in a different way [25] and formation of the faceting heaps at high temperatures is a process competing with appearance of real pyramidal huts which arise due to formation of the 16-dimer nuclei on tops of WL patches [11,25,26]. Yet, further evolution of the Ge heaps into finalized faceted clusters, such as domes, in course of Ge deposition is not excluded [24].

**Figure 3:**
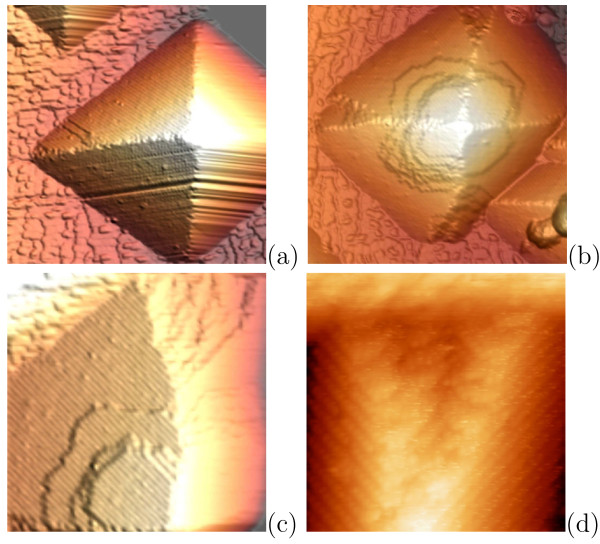
**STM empty-state images of high-temperature pyramids. ***T*_gr_=650℃; **(a) **87×87 nm, steps of the incomplete upper left facet, running normal to the base side, are seen near the left corner of the pyramid; **(b) **87×87 nm, a cluster with edges split near the base and an apex formed by a set of incomplete {105} facets; **(c) **57×57 nm, a magnified image of a facet with several {105} incomplete facets near an apex; **(d) **22×22 nm, a split edge near a base.

During further growth at high temperatures, pyramids reach large sizes becoming much greater than their low-temperature counterparts and usually form incomplete facets or split edges (Figure 3). An incomplete facet seen in Figure 3a and especially a “pelerine” of multiple incomplete facets seen in Figure 3b,c around the pyramid top indicate unambiguously that this kind of clusters grow from tops to bottoms completing facets rather uniformly from apexes to bases, and bottom corners of facets are filled the latest. Sometimes it results in edge splitting near the pyramid base (Figure 3b,d).

*RHEED* has allowed as to carry our *in-situ* explorations of forming cluster arrays. We have compared RHEED patterns of Ge/Si(001) surfaces during Ge deposition at different temperatures and a dynamics of diffraction patterns during sample heating and cooling.

**Figure 4:**
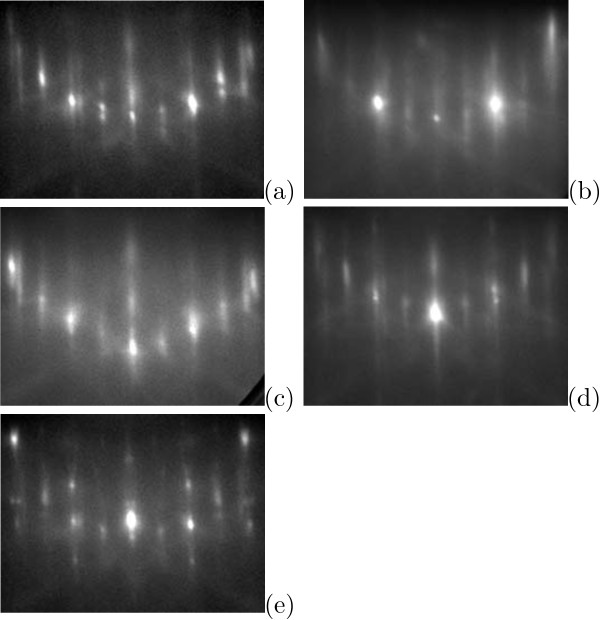
***In situ *RHEED patterns of Ge/Si(001) films. ***E* = 10 keV, [110] azimuth; (**a**) *T*_gr_= 650℃, *h*_Ge_= 4 Å; (**b**) *T*_gr_= 360℃, *h*_Ge_= 4 Å; (**c**) *T*_gr_= 650℃, *h*_Ge_= 5 Å; (**d**) *T*_gr_= 650℃, *h*_Ge_= 5 Å, annealing at the deposition temperature for 7 min; (**e**) *T*_gr_= 650℃, *h*_Ge_= 6 Å, the similar pattern is obtained for *T*_gr_= 600℃; the patterns were obtained at room temperature after sample cooling.

Diffraction patterns of reflected high-energy electrons for samples of thin (*h*_Ge_= 4 Å) Ge/Si(001) films deposited at high (650 or 600℃) and low (360℃) temperatures with equal effective thicknesses are presented in Figure 4a,b. The patterns are similar and represent a typical (2×1) structure of Ge WL; reflexes associated with appearance of huts (the 3D-reflexes) are absent in both images, that agrees with the data of the STM analysis. Diffraction patterns presented in Figure 4a,c,e are related to the samples with *h*_Ge_ increasing from 4 to 6 Å. The 3D-reflexes are observed only in the pattern of the samples with *h*_Ge_= 6 Å, that is also in good agreement with the STM data [12,26].

Influence of the sample annealing at the deposition temperature is illustrated by a complimentary pair of the RHEED patterns given in Figure 4c,d. Annealing of specimens at the temperature of growth (650℃) resulted in appearance of the 3D-reflexes (Figure 4d) that also corresponds with the results of our STM studies [12].

**Figure 5:**
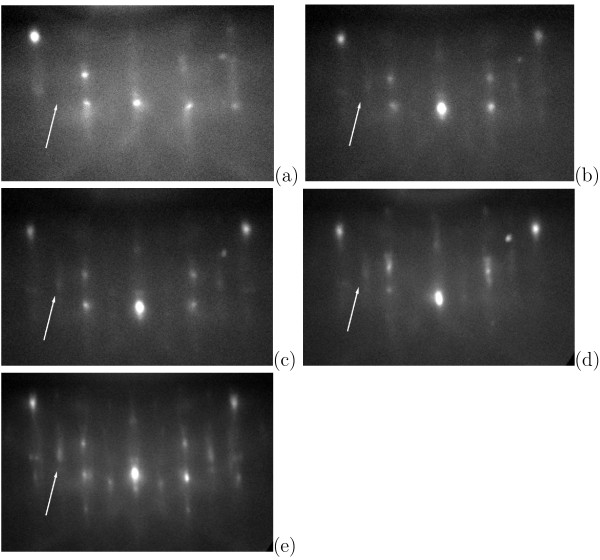
**RHEED patterns of Ge/Si(001) deposited at 600℃ obtained during sample cooling.*** h*_Ge_= 6 Å; *E* = 10 keV, [110] azimuth; cooling rate is ∼0.4℃/s (see the cooling diagram in Ref. [10]); **(a) ***T*=600℃, before cooling; (b)–(d) during cooling, time from beginning of cooling (min.): **(b)** 1, **(c)** 2, **(d)** 3; **(e)** room temperature, after cooling; arrows indicate the arising ½-reflexes to demonstrate a process of the (2×1) pattern appearance; the images were cut from frames of a film.

Difference in evolution of diffraction patterns during the deposition of Ge is a characteristic feature of the high-temperature mode of growth in comparison with the low-temperature one. The initial Si(001) surface before Ge deposition is (2×1) reconstructed. At high temperatures, as *h*_Ge_ increases, diffraction patterns evolve as (2×1)→(1×1)→(2×1) with very weak ½-reflexes. Brightness of the ½-reflexes gradually increases (the (2×1) structure becomes pronounced) and the 3D-reflexes arise only during sample cooling (Figure 5). At low temperatures, the RHEED patterns change as (2×1)→(1×1)→(2×1)→(2×1) + 3D-reflexes. The resultant pattern does not change during sample cooling.

This observation reflects the process of Ge cluster “condensation” from the 2D gas of mobile Ge adatoms. High Ge mobility and low cluster nucleation rate in comparison with fluxes to competitive sinks of adatoms determines the observed difference in the surface structure formation at high temperatures as compared with that at low temperatures [12,24] when the adatom flux to nucleating and growing clusters predominates and adatom (addimer) mobility is relatively small.

#### STM and HRTEM study of Ge/Si heterostructures with QD array: morphology and defects

Structures overgrown with Si were examined by means of HRTEM for structural perfection or possible defects, e.g., imperfections induced by array defects reported in Ref. [29].

**Figure 6:**
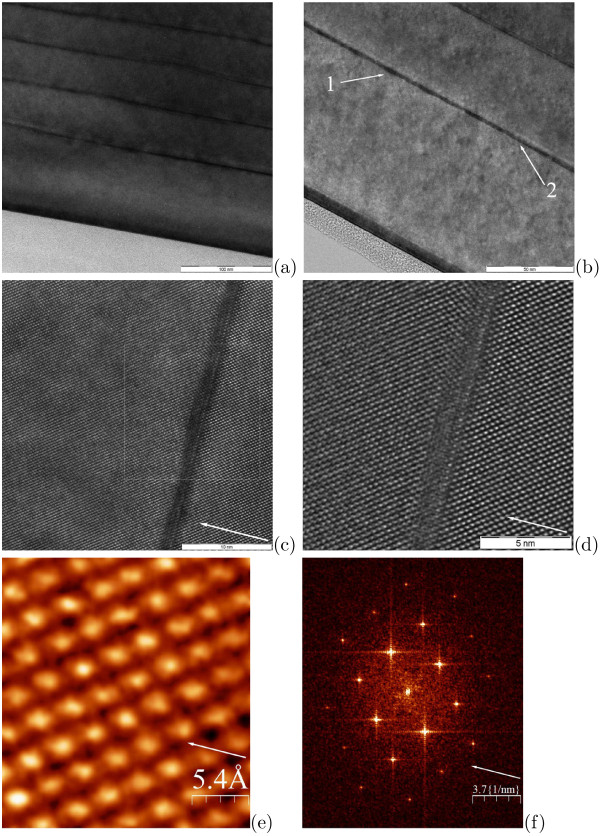
**HRTEM data for the five-layer Ge/Si heterostructure with buried Ge clusters.*** h*_*Ge*_ = 6 Å (see Figure 2a); **(a)** a long shot, the mark is 100 nm; **(b)** Ge clusters resolved in a layer, figure ‘1’ indicates one of the clusters, ‘2’ shows a WL segment; the mark is 50 nm; **(c)**,**(d)** magnified images of a Ge cluster, the panel **(d)** corresponds to the light square in the panel **(c)**; the marks are 10 and 5 nm, respectively; **(e)** a close-up image of a domain next to the top of the cluster imaged in **(d)**; **(f)** the Fourier transform of the image **(e)**, the measured periods are ∼5.4 Å along [001] and ∼3.8 Å along [110]; arrows in panels **(c)** to **(f)** indicate the [001] direction.

Data of HRTEM studies evidence that extended defects do not arise at low *h*_Ge_ on the buried Ge clusters and perfect epitaxial heterostructures with quantum dots form under these conditions that enables the formation of defectless multilayer structures suitable for device applications. Figure 6 relates to the five-layer Ge/Si structure with *h*_*Ge*_ = 6 Å. We succeeded to resolve separate Ge clusters whose height is, according to our STM data [9,12], ≲ 3 ML over WL patches (Figure 6a,b). A lattice structure next to the cluster apex is not disturbed (Figure 6c,d); its parameters estimated from the Fourier transform of an image taken from this domain (Figure 6e,f), ∼5.4 Å along the [001] direction and ∼3.8 Å along [110], within the accuracy of measurements coincide with the parameters of the undisturbed Si lattice.

**Figure 7:**
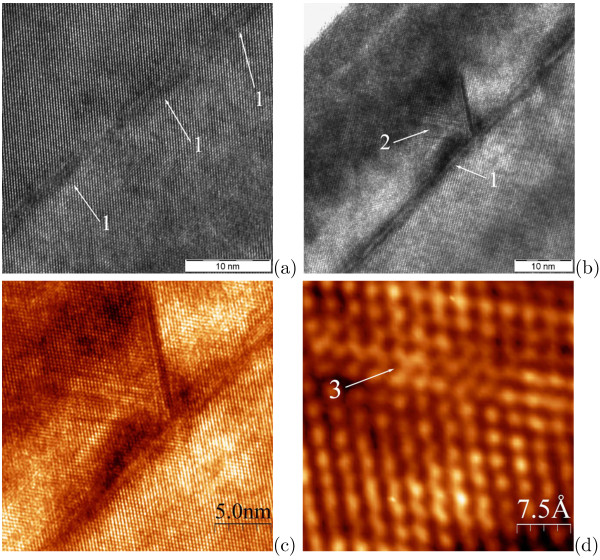
**HRTEM images of the one-layer Ge/Si structures with buried Ge clusters. ***h*_*Ge*_ = 10 Å (see Figure 2c); **(a)** a perfect epitaxial structure of Ge and Si layers; the mark is 10 nm; **(b)**, **(c)** a huge cluster (> 3,5 nm high) gives rise to tensile strain generating point and extended defects in the Si cap, the stress field spreads under the cluster [the mark is 10 nm in (b)]; **(d)** a magnified image obtained from the tensile domain, extended defects are seen; ‘1’ denotes Ge clusters, ‘2’ is a domain under tensile stress, ‘3’ indicates a twin boundary.

Stacking faults (SF) have been found to arise above Ge clusters at *h*_Ge_ as large as 10 Å (Figure 7). SFs often damage Si structures with overgrown Ge layers at this values of *h*_Ge_. A high perfection structure is observed around Ge clusters in Figure 7a although their height is up to 1.5 nm over WL (the typical height of huts is known from both our STM and HRTEM data). Yet, a tensile strained domain containing such extended defects as SFs and twin boundaries forms over a cluster shown in Figure 7b,c (twinning is clearly observable in Figure 7d). One can see, however, that this cluster is extraordinary high: its height over WL exceeds 3.5 nm. Such huge clusters have been described by us previously as defects of arrays [29]; we predicted in that article such formations to be able to destroy Ge/Si structures generating high stress fields in Si spacer layers and, as a consequence, introducing extended defects in device structures. As seen in Figure 7b,c, the stress field spreads under the cluster in the Si buffer layer grown at much higher temperature than the cap. Unfortunately, the huge Ge hut clusters (as we showed in Ref. [29], they are not domes) usually appear in the arrays and their number density was estimated as ∼10^9^ cm^−3^ from the STM data.

**Figure 8:**
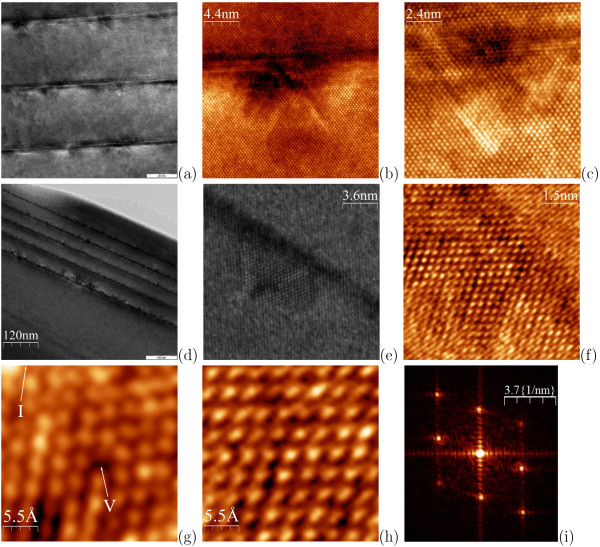
**TEM data for the five-layer Ge/Si heterostructures, *T*_*gr*_ = 360℃.** (a) to (c) *h*_*Ge*_ = 9 Å; (d) to (i) *h*_*Ge*_ = 10 Å; **(a)** domains of tensile strain in Si over Ge clusters are observed more or less distinctly near most clusters, but not around all; the surface is down; the mark equals 20 nm; **(b)**, **(c)** zoom in two strained domains, no extended defects are observed; **(d)** strained domains are more pronounced, the strain is well recognized even under some clusters; **(e)** a magnified image of a strained domain; a strained lattice is well contrasted with the normal one; **(f)** zoom in the dilated lattice, a perfectly ordered lattice is observed; **(g)** a Si domain next to the Ge/Si interface near the cluster apex, a vacancy (‘V’) and disordered lattice (upper right corner) are revealed; letter ‘I’ indicates the direction to the interface along <11 $\bar{1}$>; **(h)** the same as in **(g)** but some farther from the interface, the lattice is perfect; **(i)** the Fourier transform of an image obtained from a strained domain demonstrates an enhanced lattice parameter (the strain varies from domain to domain, the estimated lattice period in the [001] direction sometimes reaches ∼ 5.6 Å).

Strain domains are also seen next to Ge clusters in the five-layer structures depicted in Figure 8 (*h*_*Ge*_ = 9 or 10 Å). We found that such domains are not inherent to all cluster vicinities but only to some of them (Figure 8a,d). The disturbed strained domains give a contrast different from that of the undisturbed Si lattice (Figure 8e). Zoom-in micrographs of the disturbed regions show perfect order of atoms in the crystalline lattice (Figure 8b,c,e,f) everywhere except for the closest vicinities of the Si/Ge interface where point defects and a visible lattice disordering immediately next to the cluster are registered (Figure 8b,c,e,f). However, some farther from the interfaces but still near cluster apexes the crystalline order restores (Figure 8h). We have estimated the lattice parameter in the disturbed regions from the Fourier transforms of the HRTEM micrographs taken in these domains (Figure 8i). The values we obtained appeared to vary for different regions. Yet, they usually appreciably exceeded the Si lattice parameter. Moreover, they often reached the Ge parameter of ∼ 5.6–5.7 Å (along [001] and ∼ 4 Å along [110]). This might be explained either by appreciable diffusion of Ge from clusters (previously, we have already reported an appreciable diffusion of Si in Ge clusters in analogous structures from covering Si layers grown at 530℃ [30,31]) or by Si lattice stretching under the stress. Likely both factors act.

It is worth while emphasising that the stretched domains usually do not contain extended defects, as it is seen from the HRTEM micrographs, except for the cases of array defects (huge clusters) like that demonstrated in Figure 7. We suppose that the extended defects in these regions arise because the strain exceeds an elastic limit near huge clusters.

**Figure 9:**
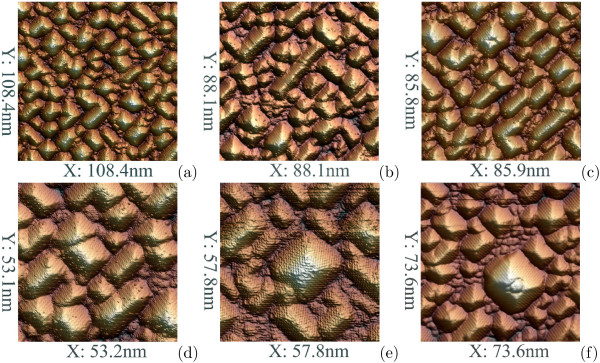
**STM images of Ge/Si(001),*** h*_*Ge*_ = 9 Å, *T*_*gr*_** = 360℃. (a)** to **(d)** array top views with different magnifications; **(e)** a large cluster in the array, ∼ 2,5 nm high; **(f)** a huge cluster (> 3,5 nm high) interpreted as an array defect.

Finally, we have tried to find out if huge clusters exist in arrays of *h*_Ge_=9 Å (Figure 9). We have been convinced that even in rather uniform arrays large clusters (Figure 9e), which might generate considerable stress, are abundant and even huge ones (Figure 9d), which should produce lattice disordering (extended defects), are available. Effect of such defects as huge clusters on device performance and a cause of their appearance in hut arrays await further detailed studies.

### Photo-emf of Ge/Si *p–i–n*-structures

#### Photo-emf spectra

We have investigated heteroepitaxial Si *p–i–n*-diodes with multilayer stacks of Ge/Si(001) QD dense arrays built in intrinsic domains and found them to exhibit the photo-emf in a wide spectral range from 0.8 to 5 *μ*m [32,33]. An effect of wide-band irradiation by infrared light on the photo-emf spectra has been observed. Here we describe the most representative data obtained for two radically different structures denoted as R 163 and R 166 (Figure 1).

**Figure 10:**
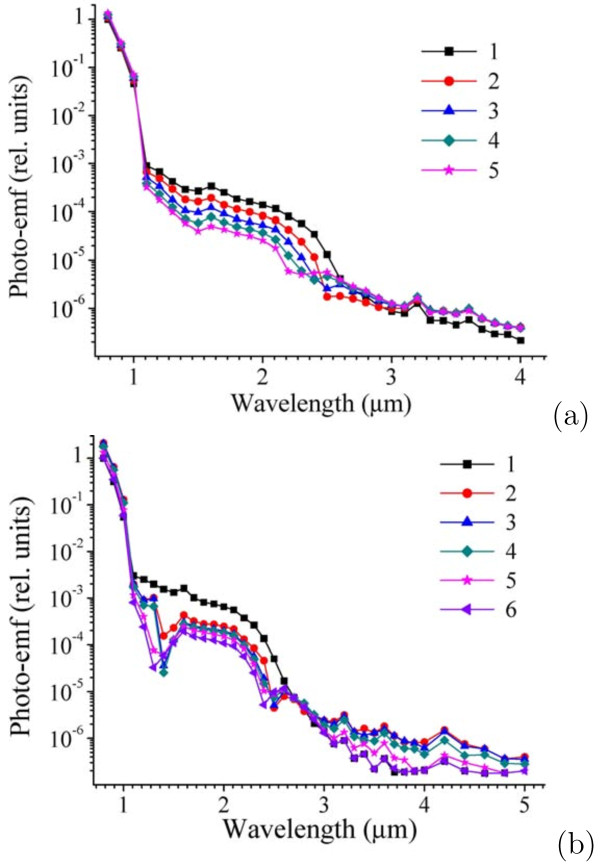
**Photo-emf spectra of the *p–i–n* structures. (a)** R 163: (1) without bias lighting; (2)–(5) under bias lighting (Ge filter): (2) *W*=0.25 mW/cm^2^; (3) *W*=0.77 mW/cm^2^; (4) *W*=1.5 mW/cm^2^; (5) *W*=2.16 mW/cm^2^; **(b)** R 166: (1) without bias lighting; (2)–(6) under bias lighting (Si filter): (2) *W*=0.63 mW/cm^2^; (3) *W*=3.3 mW/cm^2^; (4) *W*=5.3 mW/cm^2^; (5) *W*=12 mW/cm^2^; (6) *W*=17.5 mW/cm^2^.

Typical photo-emf spectra obtained for R 163 and R 166 structures are presented in Figure 10. In the spectra, we mark out three characteristic ranges which differently respond to bias lighting and differently depend on its power. 

(i) *Wavelength range from 0.8 to 1.0 μm*. The photo-emf response increases with the increase in the bias lighting power, reaches maximum at *P*≈0.63 mW/cm^2^ with a Si filter and at *P*≈2.6 mW/cm^2^ with a Ge filter and decreases with further increase in the power.

(ii) Wavelength range from 1.1 to 2.6 *μm*. The photo-emf response decreases monotonously in this range with the increase in the power of the bias lighting with any, Si or Ge, filter.

(iii) *Wavelength range > 2.6 μm*. The photo-emf response increases with the increase in the bias lighting power and comes through its maximum at *P*≈0.63 mW/cm^2^ if a Si filter is used and at *P*≈0.25 mW/cm^2^ for a Ge filter. The response decreases with further growth of the bias lighting power for a Si filter and remains unchanged when Ge filter is utilized.

**Figure 11:**
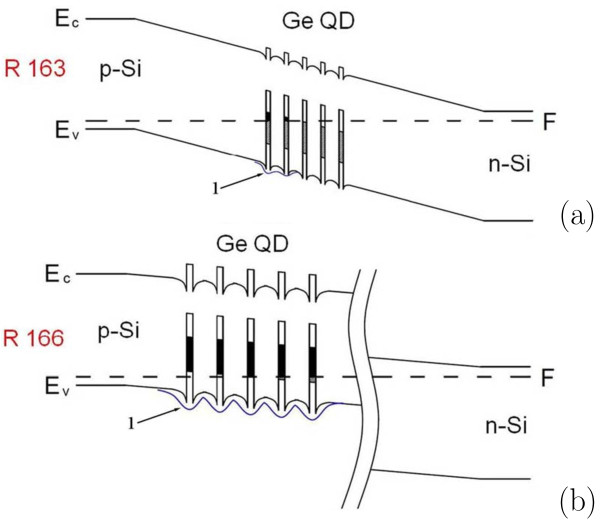
**Schematics of band structures of the Si*p–i–n*-diodes with Ge QD arrays represented in Figure **1**. (a)** R 163, **(b)** R 166; figures ‘1’, arrows and wavy lines indicate potential barriers for holes in the valence band (_*E*v_) which are associated with the Ge QD arrays; black and shaded parts of potential wells show bands of filled (above the Fermi level *F*) and empty (below *F*) energy levels in the QD ensemble; two vertical wavy lines in panel (b) indicate a gap in the drawing of a very thick buffer layer of Si (1690 nm) deposited on the n-Si substrate.

We propose the following model for explanation of these observations: In the studied structures, all QD layers are located in the *i*-domain (Figure 11). One can see from these sketches that some QD layers are positively charged (the ground states of QDs is above the Fermi level and hence they are filled by holes) while others are neutral (the QDs’ ground states are below Fermi level and hence empty). Then, one may consider a QD layer as a single ensemble of interacting centers because the average distance between QDs’ apexes is about 13 nm whereas QDs’ bases adjoin. Consequently, one can imagine an allowed energy band with some bandwidth, determined by QDs’ sizes and composition dispersion, and a certain density of states in this band. Let us explore in detail every range of the photo-emf spectra taking into account the proposed model.

#### Wavelength range from 0.8 to 1.0 *μ*m

Without bias lighting, all radiation in the Si fundamental absorption range can be believed to be absorbed in Si (cap-layer, spacers, buffer layer and substrate) and QDs are not involved in the absorption, so the total charge of QD layers remains unaltered. Electron-hole pairs are generated in the intrinsic region of the *p–i–n*-diode as a result of the absorption and separated by the junction field which converts the radiation to emf. However, carrier separation is hindered because of presence of the potential barriers for holes in the valence band which are produced by the charged QD layers situated in intrinsic domain. Calculated height of these barriers equals 0.1 to 0.2 eV depending on the layer position in the structure.

Transitions from QD ensemble states to the valence and conduction bands of Si start under bias lighting. Carriers excited by bias lighting do not contribute to the photo-emf signal measured at the modulation frequency of the narrow-band radiation. QDs captured a photon change their charge state. An effective layer charge decreases as a result of the absorption of the bias lighting radiation that results in reduction of potential barrier height and more efficient carrier separation in the junction field. Increase in the photo-emf response in the fundamental absorption range under bias lighting is explained by this process.

#### Wavelength range from 1.1 to 2.6 *μ*m

This band is entirely below the Si fundamental absorption range. Therefore the response in this region cannot be explained in terms of absorption in bulk Si. One can explain the presence of the photo-emf signal in this region considering the following model: Both hole transitions from the QD ensemble states to the valence band and electron transitions from the QD ensemble states to the conduction band due to absorption of photons with the energy between ∼1.12 and ∼0.4 eV are possible. The probability of every kind of the transitions is determined by the photon energy, the density of states in the QD ensemble and by effective charge of the QD layer.

It follows from theoretical studies [34,35] and experiments on photoluminescence [36,37] that photons with energies ranged from 0.7 to 0.9 eV are required for electron transitions from the QD states to the conduction band. However, it is necessary to mention the research of photoconductivity [38], in which electron transitions for low photon energy (∼0.4 eV) have been shown to be likely. The availability of these transitions is explained by dispersion of sizes and composition of QDs, effect of diffusion on the hetero-interface and deformation effects.

The likelihood of electron transitions drops rapidly with photon energy decrease because of reduction of the density of states in the QDs ensemble when approaching to the conduction band edge. This is the reason of the observed monotonous decrease in the photo-emf signal with the increase in the radiation wavelength in this range.

At the same time, bias lighting switching on leads to growth of concentration of the unmodulated (“dark”) carrier, depletion of QDs and as a consequence to the observed reduction of the photo-emf response at the chopping frequency.

#### Wavelength range > 2.6 *μ*m

As mentioned above, electron transitions can happen at low energy of the exciting radiation (∼ 0.4 eV) which correspond to wavelength of ∼ 3.1 *μ*m. Yet, the photo-emf signal is observed at the radiation wavelengths up to 5 *μ*m in our measurements. The presence of the photo-emf response in this range can only be explained if the QD layer is considered as a single ensemble of mutually interacting centers. An effective positive charge in the QD layer forms a potential well for electrons in the conduction band. This leads to reduction of energy needed for electron transitions from the QD ensemble states to the conduction band. Partial emptying of the states makes electron transitions possible and, at the same time, does not lead to significant change in the potential wells depth. As a result, electron transitions can happen at the exciting radiation energies as low as 0.25 eV. Hole transitions also can happen at these energies via a large number of excited states in the QD ensemble.

It may be concluded that the likelihood of the electron transitions decreases faster than that of the hole transitions as the exciting radiation energy decreases in the considered wavelength range. However, first it is necessary to empty the levels by the electron transitions to make possible hole transitions. This could be achieved by using an additional radiation of the spectral domain where the probability of the electron transitions is high. So, bias lighting stimulates the hole transitions by exciting electrons that leads to emptying the levels. In this case the electron concentration is not modulated as distinct from the hole concentration which is modulated at the chopper frequency. This explains the observed low magnitude of the photo-emf in the wavelength range > 2.6 *μ*m and its increase under bias lighting.

#### Influence of buffer layer thickness on photo-emf spectra

**Figure 12:**
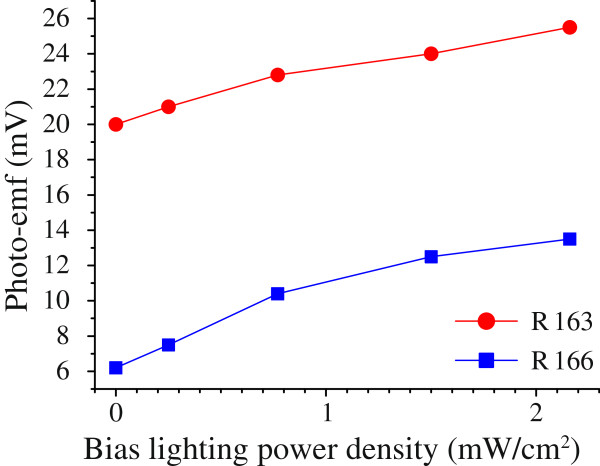
**Dependence of photo-emf response of the R 163 and R 166 *p–i–n*-structures on bias lighting power density.** The experimental conditions are the same as in Figure 10.

As seen from Figure 11, the buffer layer thickness determines the QD layers position the in intrinsic domain and thus controls the relative position of the Fermi level and the mini-band of the QD array in the region where the QD layers are situated. The charge of the QD layer is determined by the band occupation of the QD ensemble which, in turn, is controlled by the Fermi level location. For this reason the effect of bias lighting on photo-emf generated by the narrow-band radiation in the fundamental absorption range is much stronger for the R 166 structure, which have a thick buffer layer, than for the R 163 one. This is clearly seen in Figure 12. The absolute value of photo-emf in the R 166 structure is lower than that in the R 163 sample due to higher potential barriers for holes in the valence band. Yet, the photo-emf response increases with the growth of the bias lighting power much stronger in the R 166 *p–i–n*-diode than in the R 163 one.

#### Prospective photovoltaic IR detectors

On the basis of our results on the photo-emf in the Si *p–i–n*-structures with Ge quantum dots, we have recently proposed [39] a new design of photovoltaic quantum dot infrared photodetectors which enables detection of variations of photo-emf produced by the narrow-band radiation in the Si fundamental absorption range (a reference beam) under the effect of the wide-band IR radiation inducing changes in the Coulomb potential of the quantum dot ensemble which, in turn, affects the efficiency of the photovoltaic conversion of the reference beam. The quantum dot array resembles a grid of a triode in these detectors which is controlled by the detected IR light. The reference narrow-band radiation generates a potential between anode and cathode of this optically driven quantum dot triode; a magnitude of this voltage depends on the charge of the QD grid (Figure 11). Such detectors can be fabricated on the basis of any appropriate semiconductor structures with potential barriers, e.g., *p–i–n*-structures, *pn*-junctions or Schottky barriers, and built-in arrays of nanostructures.

There are many ways to deliver the reference beam to the detector, e.g., by irradiating the sensor by laser or LED. We propose, however, surface plasmon polaritons delivered to the detector structures by the plasmonic waveguides [40,41] to be applied as the reference beams in the detector circuits. This approach makes such detectors, if based on Si, fully compatible with existing CMOS fabrication processes [42] that, in turn, opens a way to development of plasmonic IR detector arrays on the basis of the monolithic silicon technology.

### THz conductivity of multilayer Ge/Si QD arrays

**Figure 13:**
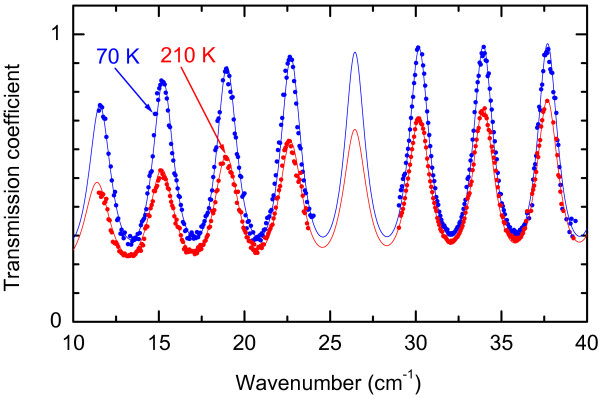
**Spectra of transmission coefficient of a silicon substrate (a commercial wafer, *ρ*=12 Ω cm), measured at two temperatures using two different BWO working in spectral ranges from 11 cm^−1^ to 24 cm^−1^ and from 29 cm^−1^ to 39 cm^−1^.** Dots show the measurement results, lines are least-square fits based on the Drude conductivity model, as described in the text.

**Figure 14:**
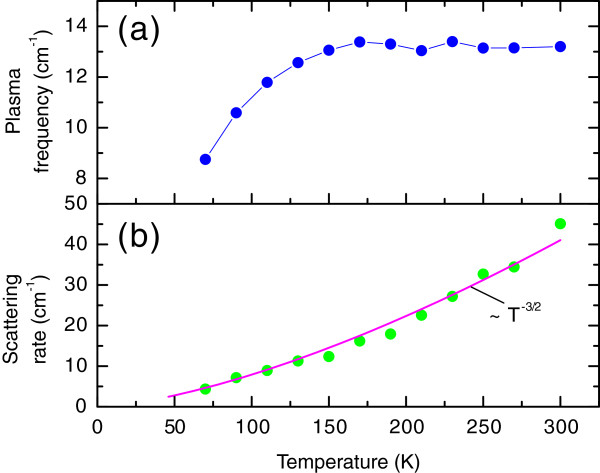
**Temperature dependences of the silicon substrate parameters obtained by fitting the transmission coefficient spectra as shown in Figure **13** and described in the text. (a)** plasma frequency of charge carriers and **(b)** scattering rate. Solid line in (b) shows the *T*^−3/2^ behavior.

The effective dynamic conductivity of Ge quantum dot layer was determined by measuring the transmission coefficient spectra of heterostructures grown on Si(001) substrates. Characteristics of the substrates were determined beforehand as demonstrated by Figure 13 and Figure 14. In Figure 13, the interferometric pattern in the transmission coefficient spectrum *Tr*(*ν*) of a plane-parallel Si substrate is clearly seen. Pronounced dispersion of *Tr*(*ν*) peaks and their temperature dependence allow to extract the parameters of the charge carriers (holes) by fitting the spectra with Eq. (2) and by modelling the sample properties with the Drude conductivity model where the complex AC conductivity is given by an expression [23,43]

(4) $$\sigma^{*}(\nu )=\sigma_{1}(\nu )+i\sigma_{2}(\nu )=\frac{\sigma_{0}\gamma^{2}}{\gamma^{2}+\nu^{2}}+i\frac{\sigma_{0}\nu \gamma }{\gamma^{2}+\nu^{2}}.$$

Here *σ*_1_ is the real part and $\sigma_{2}=\nu (\varepsilon_{\infty }-\varepsilon^{\prime })/2$ is the imaginary part of the conductivity, $\varepsilon_{\infty }$ is the high-frequency dielectric constant, $\sigma_{0}=\nu_{\mathrm{pl}}^{2}/2\gamma$ is the DC conductivity, $\nu_{\text{pl}}^{2}={\left(ne^{2}/\pi m^{*}\right)}^{\frac{1}{2}}$ is the plasma frequency of the carriers condensate, *n*, *e* and *m*^∗^ are, respectively, their concentration, charge and effective mass and *γ* is their scattering rate. Figure 14 shows the temperature variation of the plasma frequency and the scattering rate of charge carriers. Lowering of the plasma frequency is mainly connected with the carriers’ freezing out and the *γ*(*T*) behaviour is well described by a $T^{-\frac{3}{2}}$ dependence, as expected.

**Figure 15:**
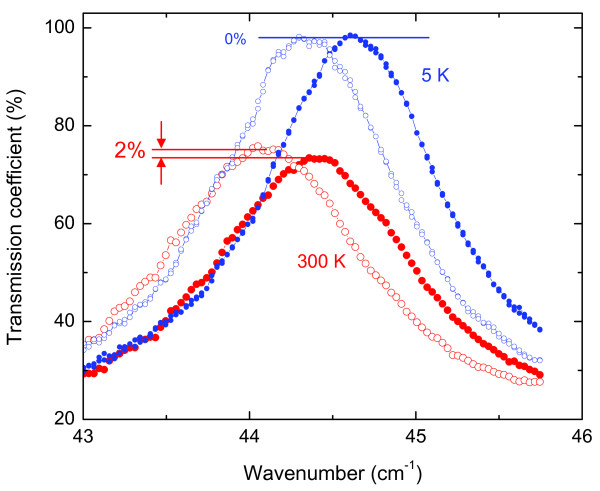
**Spectra of transmission coefficient of Ge/Si heterostructure on Si substrate (solid symbols) and of bare substrate (open symbols) measured at two different temperatures.** Horizontal lines show the difference in peak transmissivity that is observed at 300 K and disappears at ∼ 170 K. The peaks positions are shifted due to slight difference in the Si substrate thickness.

**Figure 16:**
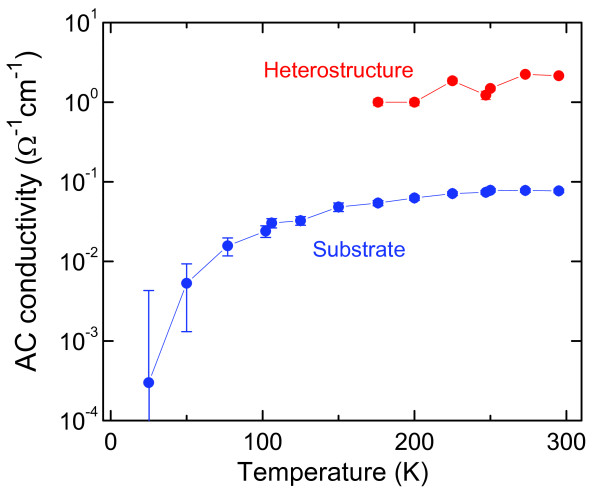
**Temperature dependences of dynamical conductivity of Ge/Si heterostructure and of Si substrate.** Frequency is around 1 THz.

The values of effective dynamical conductivity and absorption coefficient *α*=4*πk*/*λ* of the heterostructures with Ge quantum dots were determined basing on the measurements of terahertz transmission coefficient spectra of the Si substrate with the heterostructure on it as compared to the spectra of the same substrate with the heterostructure etched away; this allowed us to avoid influence of (even slight) differences in dielectric properties of substrates cut of a standard commercial silicon wafer. By comparing the so-measured transmissivity spectra we reliably detect, although small, changes in the amplitudes of interference maxima of a bare substrate caused by heterostructures. This is demonstrated by Figure 15: at *T*=300 K we clearly and firmly register a 2% lowering of the peak transmissivity introduced by the heterostructure. When cooling down, the difference decreases and we were not able to detect it below about 170 K, see Figure 16. Correspondingly, as is seen in Figure 16, the AC conductivity of the heterostructure decreases while cooling, along with the conductivity of the Si substrate. The latter observation might be an indication of the fact that the charges are delivered into the quantum dot array from the substrate; the statement, however, needs further exploration.

**Figure 17:**
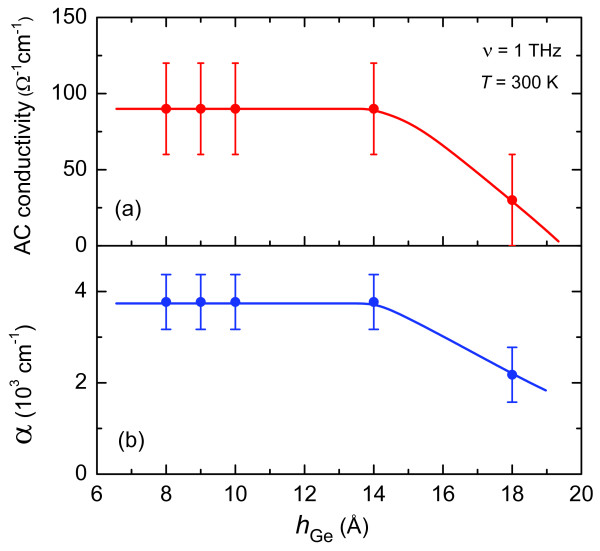
**Terahertz conductivity and absorption coefficient of Ge/Si heterostructure with Ge quantum dots versus Ge coverage. (a)** terahertz conductivity, **(b)** absorption coefficient; lines are guides to the eye.

Measuring the room temperature spectra, we have found that the AC conductivity and the absorption coefficient of the heterostructure do not depend on the effective thickness (measured by the quartz sensors during MBE) of the germanium layer (*h*_Ge_) for *h*_Ge_ ranging from 8 to 14 Å, see Figure 17. For larger coverage, *h*_Ge_>14 Å, both quantities start to decrease.

One of the main findings of this work is that the AC conductivity and absorption coefficient of Ge/Si heterostructures have been discovered to be significantly higher than those of the structure with the same amount of germanium not organized in an array of quantum dots. Crucial role played by quantum dots is supported by a decrease of _*σ*AC_ and *α* observed for large germanium coverage (*h*_Ge_ > 14 Å), when structurization into quantum dots gets less pronounced and the thickness of Ge layer becomes more uniform. On the other hand, it is worth noting that no extra absorption of terahertz radiation was detected in the samples with low coverage, *h*_Ge_=4.4 and 6 Å. This can be explained either by the absence of quantum dots in that thin Ge layer or by their small sizes, by a large fraction of the free wetting layer or by relatively large distances between the clusters as compared to their sizes, i.e., by the absence or smallness of the effect of quantum dots on the dielectric properties of the heterostructure.

As seen from Figure 17, the values $\sigma_{\text{AC}}\approx 100\;\Omega^{-1}{\mathrm{cm}}^{-1}$ and *α*≈4000 cm^−1^ are considerably higher than the values measured for bulk germanium, *σ*_AC_(Ge)≈10^−2^ Ω^−1^cm^−1^, by about four orders of magnitude, and *α*(Ge)≈40 cm^−1^, by about two orders of magnitude. Assuming that the AC conductivity of heterostructure is connected with the response of (quasi) free carriers, one can express it with a standard formula *σ*=*eμn*=*ne*^2^(2*πγm*^∗^)^−1^(*μ* is the mobility of charge carriers). Then, the observed increase has to be associated with considerable enhancement either of the mobility (suppression of scattering rate) of charge carriers within a quantum dot array or of their concentration. The second possibility has to be disregarded since the total concentration of charges in the sample (substrate plus heterostructure) remains unchanged. As far as the mobility increase is concerned, we are not aware of a mechanism that could lead to its orders of magnitude growth when charges get localized within the quantum dot array.

Another interpretation of the observed excess AC conductivity could be based on some kind of *resonance* absorption of terahertz radiation. Known infrared experiments exhibit resonances in quantum dot arrays that are caused by the transitions between quantized energy levels, as well as between the split levels and the continuum of the valence or conduction band [4,44-47]. Carriers localized within quantum dots can form bound states with the carriers in the surrounding continuum (excitons) or with optical phonons (polarons), which can in turn interact with each other and form collective complexes [3,4,45-48]. Plasma excitations generated by electromagnetic radiation in the assembly of conducting clusters or quantum dots also have energies of about 10 meV [49-51], i.e., fall into the THz band. It is important that these effects can be observed not only at low, but at elevated temperatures as well, up to the room temperature. At this stage, we are not able to unambiguously identify the origin of the THz absorption seen at *T*=170 to 300 K in Ge/Si heterostructure with Ge quantum dots. Among the aforementioned, the mechanisms involving polaritons or plasma excitations seem to be least affected by thermal fluctuations and could be considered as possible candidates. To get detailed insight into microscopic nature of the observed effect, further investigations of heterostructures with various geometric and physical parameters, as well as in a wider frequency and temperature intervals are in progress.

## Conclusions

In conclusion of the article, we highlight its main provisions.

Using high resolution STM and *in-situ* RHEED we have explored the processes of Ge hut cluster array formation and growth at low temperatures on the Ge/Si(001) wetting layer. Different dynamics of the RHEED patterns in the process of Ge hut array formation at low and high temperatures of Ge deposition reflects the difference in adatom mobility and their fluxes from 2D gas of mobile particles (atoms, dimers and dimer groups) on the surface which govern the nucleation rates and densities of arising Ge clusters.

HRTEM studies of multilayer Ge/Si heterostructures with buried arrays of Ge huts have shown that the domains of stretched lattice occurring over Ge clusters in Si layers at high Ge coverages usually do not contain extended defects. We suppose that the extended defects in these regions arise because the strain exceeds an elastic limit near huge clusters.

Silicon *p–i–n*-diodes with multilayer stacks of Ge cluster arrays built in *i*-domains have been found to exhibit the photo-emf in a wide spectral range from 0.8 to 5 *μ*m. A significant increase in photo-emf response in the fundamental absorption range under the wide-band IR radiation has been reported and explained in terms of positive and neutral charge states of the quantum dot layers and the Coulomb potential of the quantum dot ensemble. A new type of photovoltaic QDIPs is proposed in which photovoltage generated by a reference beam in the fundamental absorption band is controlled by the QD grid charge induced by the detected IR radiation [39].

Using a BWO-spectrometer, first measurements of terahertz dynamical conductivity spectra of Ge/Si heterostructures were carried out at frequencies ranged from 0.3 to 1.2 THz in the temperature interval from 5 to 300 K. The effective dynamical conductivity of the heterostructures with Ge quantum dots has been found to be significantly higher than that of the structure with the same amount of Ge not organized in quantum dots. The excess conductivity is not observed in the structures with the Ge coverage less than 8 Å. When a Ge/Si sample is cooled down the conductivity of the heterostructure decreases.

## Abbreviations

AC, alternating current; BWO, backward-wave oscillator; CMOS, complementary metal-oxide semiconductor; CZ, Czochralski or grown by the Czochralski method; DC, direct current; emf, electromotive force; HRTEM, high resolution transmission electron microscope; IR; infrared; LED, light emitting diode; MBE, molecular beam epitaxy; ML, monolayer; QD, quantum dot; QDIP, quantum dot infrared photodetector; RHEED, reflected high energy electron diffraction; SF, stacking fault; SIMS, secondary ion mass spectroscopy; STM, scanning tunneling microscope; WL, wetting layer; UHV, ultra-high vacuum.

## Competing interests

The authors declare that they have no competing interests.

## Authors' contributions

VAY conceived of the study and designed it, performed data analysis, and took part in discussions and interpretation of the results; he also supervised and coordinated the research projects. LVA participated in the design of the study, carried out the experiments, performed data analysis, and took part in discussions and interpretation of the results. MSS investigated the photo-emf spectra; he carried out the experiments, performed data analysis, and took part in discussions and interpretation of the results. VAC participated in the design of the study, took part in discussions and interpretation of the results; he also supervised the researches performed by young scientists and students. KVC took pat in the experiments on investigation of the photo-emf spectra and the terahertz conductivity; he prepared experimental samples and took part in discussions and interpretation of the results. OVU performed the HRTEM studies and took part in discussions and interpretation of the results. VPK participated in the design of the study, took part in discussions and interpretation of the results; he also supervised the research project. ESZ carried out the experiments on the terahertz spectroscopy; she performed measurements and data analysis, and took part in discussions and interpretation of the results. ASP participated in the studies by the terahertz spectroscopy; he took part in discussions and interpretation of the results. IES participated in the studies by the terahertz spectroscopy; he took part in discussions and interpretation of the results. BPG performed the explorations by the terahertz spectroscopy; he participated in the design of the study, performed measurements and data analysis, and took part in discussions and interpretation of the results; he also supervised the research project. All authors read and approved the final manuscript.

## References

[B1] Pchelyakov OP Bolkhovitjanov YB Dvurechenskiĭ AV Nikiforov AI Yakimov AI Voigtländer B Molecular beam epitaxy of silicon–germanium nanostructures Thin Solid Films 2000 367 75 10.1016/S0040-6090(00)00667-2

[B2] Pchelyakov OP Bolkhovitjanov YB Sokolov LV Nikiforov AI Yakimov AI Voigtländer B Dvurechenskiĭ AV Silicon–germanium nanostructures with quantum dots: Formation mechanisms and electrical properties Semicond 2000 34 1229 10.1134/1.1325416

[B3] Yakimov AI Dvurechenskii AV Kirienko VV Yakovlev YI Nikiforov AI Long-range Coulomb interaction in arrays of self-assembled quantum dots Phys Rev B 2000 61 10868 10.1103/PhysRevB.61.10868

[B4] Drexler H Leonard D Hansen W Kotthaus JP Petroff PM Spectroscopy of quantum levels in charge-tunable InGaAs quantum dots Phys Rev Lett 1994 73 2252 10.1103/PhysRevLett.73.2252 10057011

[B5] Lipparini E Barberan N Barranco M Pi M Serra L Far-infrared edge modes in quantum dots Phys Rev B 1997 56 12375 10.1103/PhysRevB.56.12375

[B6] Wang KL Tong S Kim HJ Properties and applications of SiGe nanodots Materials Science in Semiconductor Processing 2005 8 389 10.1016/j.mssp.2004.09.134

[B7] Wang KL Cha D Liu J Chen C Ge/Si self-assembled quantum dots and their optoelectronic device applications Proc of the IEEE 2007 95 9 1866

[B8] Yakimov AI Dvurechenskii AV Nikiforov AI Proskuryakov YY Interlevel Ge/Si quantum dot infrared photodetector J Appl Phys 2001 89 5676 10.1063/1.1346651

[B9] Arapkina LV Yuryev VA Classification of Ge hut clusters in arrays formed by molecular beam epitaxy at low temperatures on the Si(001) surface Phys Usp 2010 53 3 279 [ArXiv:0907.4770] 10.3367/UFNe.0180.201003e.0289

[B10] Arapkina LV Yuryev VA Chizh KV Shevlyuga VM Storojevyh MS Krylova LA Phase transition on the Si(001) clean surface prepared in UHV MBE chamber: A study by high resolution STM and in situ RHEED Nanoscale Res Lett 2011 6 218 [ArXiv:1009.3909] 10.1186/1556-276X-6-218 21711733 PMC3211275

[B11] Arapkina LV Yuryev VA CMOS compatible dense arrays of Ge quantum dots on the Si(001) surface: Hut cluster nu-cleation, atomic structure, and array life cycle during UHV MBE growth Nanoscale Res Lett 2011 6 345 [ArXiv:1009.3831] 10.1186/1556-276X-6-345 21711886 PMC3211434

[B12] Yuryev VA Arapkina LV Ge quantum dot arrays grown by ultrahigh vacuum molecular-beam epitaxy on the Si(001) surface: nucleation, morphology, and CMOS compatibility Nanoscale Res Lett 2011 6 522 [ArXiv:1104.2848] 10.1186/1556-276X-6-522 21892938 PMC3212061

[B13] Eltsov KN Ultrahigh vacuum scanning tunneling microscope STM GPI-300 [ http://surface.gpi.ru/papers/gpi300e.pdf]

[B14] Eltsov KN Klimov AN Kosyakov AN Obyedkov OV Shevlyuga VM Yurov VY Ultrahigh vacuum scanning tunnelling microscope GPI-300 Chemical state and atomic structure of fcc metal surfaces in chemical reaction with halogens, Volume 59 of Proc. of General Physics Institute. Edited by Konov VI, Eltsov KN 2003 Moscow, Russia, Nauka 45 45

[B15] Yurov VY Klimov AN Scanning tunneling microscope calibration and reconstruction of real image: Drift and slope elimination Rev Sci Instrum 1994 65 1551 10.1063/1.1144890

[B16] Horcas I Fernandez R Gomez-Rodriguez JM Colchero J Gomez-Herrero J Baro AM WSxM: A software for scanning probe microscopy and a tool for nanotechnology Rev Sci Instrum 2007 78 013705 10.1063/1.2432410 17503926

[B17] Reinhardt KA Kern W(Eds) Handbook of silicon wafer cleaning technology 2008 William Andrew Inc., Norwich, NY,USA

[B18] Arapkina LV Shevlyuga VM Yuryev VA Structure and peculiarities of the (8×n)-type Si(001) surface prepared in a molecular beam epitaxy chamber: A scanning tunneling microscopy study JETP Lett 2008 87 215 [ArXiv:0908.1346] 10.1134/S0021364008040085

[B19] Arapkina LV Yuryev VA Shevlyuga VM Chizh KV Phase transition between (2×1) and c(8×8) reconstructions observed on the Si(001) surface around 600°C JETP Lett 2010 92 5 310 [ArXiv:1007.0329] 10.1134/S0021364010170091

[B20] Kozlov G Volkov A Millimeter and submillimeter wave spectroscopy of solids Topics in Applied Physics, Volume 74. Edited by Grüner G 1998 Berlin Springer 51 51

[B21] Gorshunov B Volkov A Spektor I Prokhorov A Mukhin A Dressel M Uchida S Loidl A Terahertz BWO-spectroscopy Int J Infrared Millimeter Waves 2005 26 1217 10.1007/s10762-005-7600-y

[B22] Born M Wolf E Principles of Optics 1959 Pergamon, London

[B23] Dressel M Grüner G Electrodynamics of Solids 2002 Cambridge University Press, London

[B24] Yuryev VA Arapkina LV Nucleation of Ge clusters at high temperatures on Ge/Si(001) wetting layer J Appl Phys 2012 111 094307 [ArXiv:1105.6012] 10.1063/1.4707936

[B25] Arapkina LV Yuryev VA Nucleation of Ge quantum dots on the Si(001) surface Phys Rev B 2010 82 045315 [ArXiv:0907.4665]

[B26] Arapkina LV Yuryev VA An initial phase of Ge hut array formation at low temperature on Si(001) J Appl Phys 2011 109 10 104319 [ArXiv:1009.3831] 10.1063/1.3592979

[B27] Goldfarb I Hayden PT Owen JHG Briggs GAD Nucleation of “hut” pits and clusters during gas-source molecular-beam epitaxy of Ge/Si(001) in in situ scanning tunnelling microscopy Phys Rev Lett 1997 78 20 3959 10.1103/PhysRevLett.78.3959

[B28] Goldfarb I Effect of strain on the appearance of subcritical nuclei of Ge nanohuts on Si(001) Phys Rev Lett 2005 95 025501 16090695 10.1103/PhysRevLett.95.025501

[B29] Yuryev VA Arapkina LV Defects of Ge quantum dot arrays on the Si(001) surface Physica B 2009 404 4719 [ArXiv:0908.0841] 10.1016/j.physb.2009.08.118

[B30] Kucherenko IV Vinogradov VS Melnik NN Arapkina LV Chapnin VA Chizh KV Yuryev VA Effect of interdiffusion and quantum confinement on Raman spectra of the Ge/Si(100) heterostructures with quantum dots Proceedings of the 16th International Symposium “Nanostructures: Physics and Technology” 2008 Ioffe Physico-Technical Institute, St. Petersburg, Russia, Vladivostok, Russia 199 199 [ArXiv:0908.1378]

[B31] Kucherenko IV Vinogradov VS Mel’nik NN Arapkina LV Chapnin VA Chizh KV Yuryev VA The role of interdiffusion and spatial confinement in the formation of resonant Raman spectra of Ge/Si(100) heterostructures with quantum-dot arrays Phys Solid State 2008 50 1970 [ArXiv:1010.1927] 10.1134/S1063783408100302

[B32] Arapkina LV Storozhevykh MS Chapnin VA Chizh KV Uvarov OV Kalinushkin VP Yuryev VA Ge/Si(001) heterostructures with dense arrays of Ge quantum dots: Low-temperature formation, characterization by high resolution STM and TEM-, and exploration of photo-EMF spectra Second International Symposium on Nanotechnology, Energy and Space (“NES-2011”) 2011 Institute of Microelectronics Technology and High Purity Materials of the Russian Academy of Sciences, Chernogolovka, Moscow Region, Russia 32 32

[B33] Storozhevykh MS Arapkina LV Chapnin VA Kalinushkin VP Chizh KV Yuryev VA Photosensitivity of Si p–i–n-structures with Ge quantum dots and effect of IR irradiation on its magnitude Russian Conference and school on Actual Problems of Semiconductor Photoelectronics (“Photonics-2011”). Edited by Aseev AL, Dvurechenskiĭ AV 2011 Novosibirsk, Russia: A. V. Rzhanov Institute of Semiconductor Physics of Siberian Brunch of the Russian Academy of Sciences 36 36

[B34] Gerasimenko NN Parhomenko YN Silicon as a Material for Nanoelectronics 2007 Technosphera, Moscow, Russia

[B35] Brudnyi VN Grinyaev SN Dvurechenskii AV Electronic properties of silicon with ultrasmall germanium clusters Phys Solid State 2005 47 11 2020 10.1134/1.2131138

[B36] Talalaev VG Cirlin GE Tonkikh AA Zakharov ND Werner P Gösele U Tomm JW Elsaesser T Miniband-related 1.4–1.8 μm luminescence of Ge/Si quantum dot superlattices Nanoscale Res Lett 2006 1 137 10.1007/s11671-006-9004-x

[B37] Larsson M Elfving A Holtz PO Hansson G Ni WX Photoluminescence study of Si/Ge quantum dots Surf Sci 2003 532-535 832

[B38] Talochkin AB Chistokhin IB Markov VA Lateral photoconductivity of multilayer Ge/Si structures with Ge quantum dots Semicond 2009 43 8 997 10.1134/S1063782609080077 19420590

[B39] Arapkina LV Storozhevykh MS Chizh KV Chapnin VA Method of detection of electromagnetic radiation and device for its implementation Patent pending; Russian Federation Patent Application No. 2012122054 May 29, 2012

[B40] Bozhevolnyi SI Volkov VS Devaux E Laluet JY Ebbesen TW Channel plasmon subwavelength waveguide components including interferometers and ring resonators Nature 2006 440 508 10.1038/nature04594 16554814

[B41] Holmgaard T Chen Z Bozhevolnyi SI Markey L Dereux A Krasavin AV Zayats AV Bend- and splitting loss of dielectric-loaded surface plasmon-polariton waveguides Optics Express 2008 16 18 13585 10.1364/OE.16.013585 18772968

[B42] Krasavin AV Zayats AV Silicon-based plasmonic waveguides Optics Express 2010 18 11 11791 10.1364/OE.18.011791 20589040

[B43] Sokolov AV Optical Properties of Metals 1967 American Elsevier, New York

[B44] Heitmann D Kotthaus JP The spectroscopy of quantum dot arrays Phys Today 1993 46 56

[B45] Boucaud P Thanh VL Sauvage S Débarre D Bouchier D Intraband absorption in Ge/Si self-assembled quantum dots Appl Phys Lett 1999 74 401 10.1063/1.123083

[B46] Weber A Gauthier-Lafaye O Julien FH Brault J Gendry M Desieres Y Benyattou T Strong normal-incidence infrared absorption in self-organized InAs/InAlAs quantum dots grown on InP(001) Appl Phys Lett 1999 74 413 10.1063/1.123045

[B47] Sauvage S Boucaud P Gerard JM Thierry-Mieg V In-plane polarized intraband absorption in InAs/GaAs self-assembled quantum dots Phys Rev B 1998 58 10562 10.1103/PhysRevB.58.10562

[B48] Hameau S Isaia JN Guldner Y Deleporte E Verzelen O Ferreira R Bastard G Zeman J Gerard JM Far-infrared magnetospectroscopy of polaron states in self-assembled InAs/GaAs quantum dots Phys Rev B 2002 65 085316

[B49] Sikorski C Merkt U Spectroscopy of electronic states in InSb quantum dots Phys Rev Lett 1989 62 2164 10.1103/PhysRevLett.62.2164 10039872

[B50] Dahl C Kotthaus JP Nickel H Schlapp W Coulomb coupling in arrays of electron disks Phys Rev B 1992 46 15590 10.1103/PhysRevB.46.15590 10003697

[B51] Demel T Heitmann D Grambow P Ploog K Nonlocal dynamic response and level crossing in quantum-dot structures Phys Rev Lett 1990 64 788 10.1103/PhysRevLett.64.788 10042078

